# A Rare Clinical Presentation of Malignant Pleural Mesothelioma With Central Nervous System Metastases: A Case Report

**DOI:** 10.7759/cureus.92297

**Published:** 2025-09-14

**Authors:** Magdalena A Miernik-Skrzypczak, Elwira Misztela-Lisiecka, Piotr Nowakowski, Anna Strozak, Grazyna Waska

**Affiliations:** 1 General Medicine, The Lower Silesian Center of Oncology, Pulmonology and Hematology, Wrocław, POL; 2 Medicine, Pabianice Medical Centre Ltd., Pabianice, POL; 3 Internal Medicine, Municipal Hospital in Gliwice, Gliwice, POL; 4 Internal Medicine, Dr. Anna Gostynska Wolski Hospital, Warsaw, POL; 5 Internal Medicine, Specialist Hospital No. 1 in Bytom, Bytom, POL

**Keywords:** central nervous system metastases, malignant pleural mesothelioma, mesothelial neoplasm, palliative chemotherapy, palliative radiotherapy, pleural effusion, rare malignancy

## Abstract

Malignant pleural mesothelioma (MPM) is a rare and aggressive cancer arising from the pleural mesothelial cells, most often associated with long-term asbestos exposure. Symptoms are typically nonspecific, including shortness of breath, chest pain, and pleural effusion, which may delay diagnosis. We report a case of a 63-year-old woman with bilateral MPM and subsequent central nervous system (CNS) metastases. She received systemic chemotherapy with cisplatin and pemetrexed, achieving a partial radiological response. Several months after completing treatment, she developed neurological symptoms. Imaging revealed multiple CNS metastases. Palliative radiotherapy provided temporary control; however, her condition progressively deteriorated. This case illustrates an unusual presentation and metastatic pattern of MPM in a female patient, highlighting diagnostic and therapeutic challenges, as well as the aggressive nature of the disease despite palliative care.

## Introduction

Malignant pleural mesothelioma (MPM) is a rare, aggressive neoplasm originating from mesothelial cells lining the pleura. Less frequently, mesothelioma can arise from the peritoneum or pericardium, with the pericardial subtype being the rarest [1,2]. MPM accounts for the majority of mesothelioma cases and is strongly associated with asbestos exposure, which is identified in approximately 70-90% of the affected individuals [1,3]. Asbestos, a group of silicate minerals formerly widely employed in various industries for its thermal resistance, remains a significant etiological factor. Beyond occupational settings, naturally occurring asbestos in the environment constitutes an additional source of exposure. The carcinogenic process is multistage, and the probability of disease development correlates with both the duration and intensity of the exposure [3,4]. Additional risk factors include previous chest irradiation, exposure to fibrous minerals such as erionite or fluoro-edenite (found in volcanic ash), and contact with certain chemical agents. A possible association with simian virus 40 (SV40) infection has also been suggested. Molecular alterations, particularly involving tumor suppressor genes such as BAP1, NF2, CDKN2A, TP53, SETD2, and SETDB1, are increasingly recognized as contributing to mesothelioma pathogenesis [1,4-6]. Despite being the most common primary pleural malignancy, MPM remains a rare cancer, with an estimated incidence of approximately one case per 120,000 individuals [7,8]. The disease predominantly affects men over 60 years of age and carries a poor prognosis, with median survival ranging from six to 18 months following diagnosis [7,8]. MPM is often asymptomatic until advanced stages. When present, symptoms typically include progressive dyspnea, chest pain, persistent cough, and weight loss, most often resulting from pleural effusion or local tumor invasion [6-9]. In advanced disease, metastases may involve mediastinal lymph nodes or distant organs such as the liver, adrenal glands, and less commonly, the brain [2,6,9]. Chest computed tomography (CT) remains the cornerstone of diagnostic imaging, allowing for assessment of tumor spread and involvement of adjacent structures. Additional modalities such as positron emission tomography (PET) and magnetic resonance imaging (MRI) are often used for staging and treatment planning. Diagnostic confirmation requires histopathological analysis of tissue samples obtained through procedures such as bronchoscopy, mediastinoscopy, or thoracoscopy [1,2,6,7,9]. While pleural fluid cytology is frequently performed, its diagnostic yield is limited. Video-assisted thoracoscopic surgery (VATS) is considered the gold standard for obtaining adequate tissue for definitive diagnosis and molecular testing [10]. Histological diagnosis can be challenging due to tumor heterogeneity. Immunohistochemical (IHC) staining, using markers such as calretinin, Wilms’ tumor antigen 1 (WT1), and cytokeratin 5/6, is essential to differentiate MPM from metastatic adenocarcinomas or benign mesothelial proliferations [1,5,6,10]. Staging is based on the 2017 Union for International Cancer Control (UICC) TNM classification, which considers tumor extension, nodal involvement, and distant metastases [6,11]. Early-stage disease (IA-II) is potentially amenable to curative treatment, but most patients present at a stage where only palliative therapy is feasible [6,7]. For select patients, radical surgical resection offers the possibility of long-term disease control. Surgical options include extrapleural pneumonectomy (EPP) and pleurectomy/decortication (P/D), the latter currently favored due to lower postoperative morbidity [6,7,11,12]. Surgery is often combined with chemotherapy and/or radiotherapy, which can be administered in neoadjuvant, adjuvant, or intraoperative settings [6,7]. The combination of cisplatin and pemetrexed remains the standard first-line systemic regimen for unresectable MPM [6,7,12]. Bevacizumab may further improve outcomes when added to chemotherapy. In patients with non-epithelioid subtypes, immune checkpoint inhibitors such as nivolumab and ipilimumab have demonstrated clinical benefit [2,7,12]. Patients ineligible for disease-modifying therapy receive palliative treatment aimed at symptom control and quality of life improvement. Procedures such as talc pleurodesis, indwelling pleural catheter insertion, or subtotal pleurectomy may be used to manage malignant pleural effusion [7]. This case was previously presented as a poster at the 3rd Student Conference on Palliative Medicine, organized by the Medical University of Gdańsk and held online on April 9, 2022.

## Case presentation

A 63-year-old female patient under intensified oncologic surveillance due to a germline breast cancer gene 1 (BRCA1) mutation and a significant family history of cancer presented to the emergency department with low-grade fever, chest pain, and resting dyspnea. She reported no history of occupational or environmental exposure to asbestos, radiation, or other known carcinogens. Physical examination revealed absent breath sounds and dullness to percussion over the right hemithorax. Chest radiography (CXR) demonstrated a right-sided pleural effusion. The patient was admitted to the Pulmonology Department, where multiple thoracenteses were performed. Pleural fluid was sent for cytological and histopathological analysis. Cytology revealed malignant cells consistent with adenocarcinoma, while Serratia marcescens was isolated from bronchoalveolar lavage. Antibiotic therapy was adjusted according to culture sensitivity. Due to the presence of malignant cells, VATS was performed, and biopsies were obtained. Within one month, a left-sided pleural effusion developed, prompting left-sided pleuroscopy with talc pleurodesis.

Histopathology and IHC staining confirmed bilateral epithelioid MPM. The patient was referred to an oncology center and deemed eligible for palliative systemic chemotherapy with cisplatin and pemetrexed. She completed six cycles administered over 18 weeks, achieving a partial radiological response on follow-up CT. Seven months after completing chemotherapy, the patient developed neurological symptoms including headaches, ataxia, diplopia, and dizziness. Brain CT revealed multiple metastases. She was referred for palliative cranial radiotherapy. Despite transient radiological stabilization on imaging, her condition deteriorated clinically. Eighteen months after initial symptom onset and one month following radiotherapy, the patient lapsed into a coma and died within a week (Table 1).

**Table 1:** Timeline of the clinical course in a patient with bilateral malignant pleural mesothelioma and CNS metastases VATS: video-assisted thoracoscopic surgery; CNS: central nervous system; IHC: Immunohistochemical.

Month from symptom onset	Clinical event/ Intervention
0	Onset of symptoms: low-grade fever, chest pain, resting dyspnea. Chest X-ray: right-sided pleural effusion; hospitalization and multiple thoracenteses
1	VATS and biopsy of right pleura; development of left-sided effusion → pleuroscopy with talc pleurodesis
1-2	Histopathology and IHC: bilateral epithelioid malignant pleural mesothelioma
3-8	Palliative chemotherapy (cisplatin + pemetrexed), six cycles → partial radiological response
15	Neurological symptoms: headaches, ataxia, diplopia, dizziness; Brain CT: multiple CNS metastases
16-17	Palliative cranial radiotherapy
17-18	Clinical deterioration → coma
18	Patient death

## Discussion

This case represents a rare clinical presentation of bilateral MPM with central nervous system (CNS) metastases. While pleural mesothelioma typically presents with unilateral involvement, bilateral disease, as in this case, can resemble metastatic pleural involvement from extrapulmonary primaries [1,5,9]. Furthermore, the cytological appearance of adenocarcinoma-like cells highlighted diagnostic challenges and emphasized the essential role of IHC in accurate differential diagnosis [9]. The presence of a BRCA1 mutation, typically linked to breast and ovarian cancers, suggests broader oncogenic susceptibility, possibly including mesothelioma [3,4,12]. Although not definitively linked to MPM, the mutation may reflect underlying genomic instability contributing to mesothelial carcinogenesis. 

CNS metastases in MPM are rare but increasingly recognized due to improved systemic control and longer patient survival [1,7]. Median survival following CNS involvement is typically under four months [11]. Our patient survived approximately three months from the diagnosis of CNS metastases and 18 months from symptom onset. Initial treatment with cisplatin and pemetrexed elicited a meaningful response, suggesting a relatively indolent tumor biology. However, the eventual CNS progression underscores the limitations of current treatment options for disseminated MPM. 

This case highlights the limitations of current treatment modalities in MPM, particularly when the disease disseminates beyond the thoracic cavity. It emphasizes the importance of vigilant follow-up, especially for atypical metastatic presentations. Future therapeutic approaches should explore the integration of immunotherapy or targeted molecular agents.

## Conclusions

MPM remains a formidable clinical challenge due to its aggressive behavior and limited therapeutic options. Despite advances in multimodal treatment strategies, overall prognosis remains unfavorable, and curative options are limited to patients eligible for surgery combined with systemic and/or radiation therapy. Early detection, accurate histopathological assessment, and timely referral to specialized centers are essential for optimizing management and improving patient outcomes. 

This case underscores the need for heightened clinical suspicion in atypical presentations, the importance of comprehensive diagnostic evaluation, and the critical role of multidisciplinary care. It also highlights the importance of further research into novel systemic therapies, including immunotherapy and targeted agents, which may improve survival and quality of life. CNS involvement, though rare, may occur in prolonged survivors and should be considered during follow-up.
